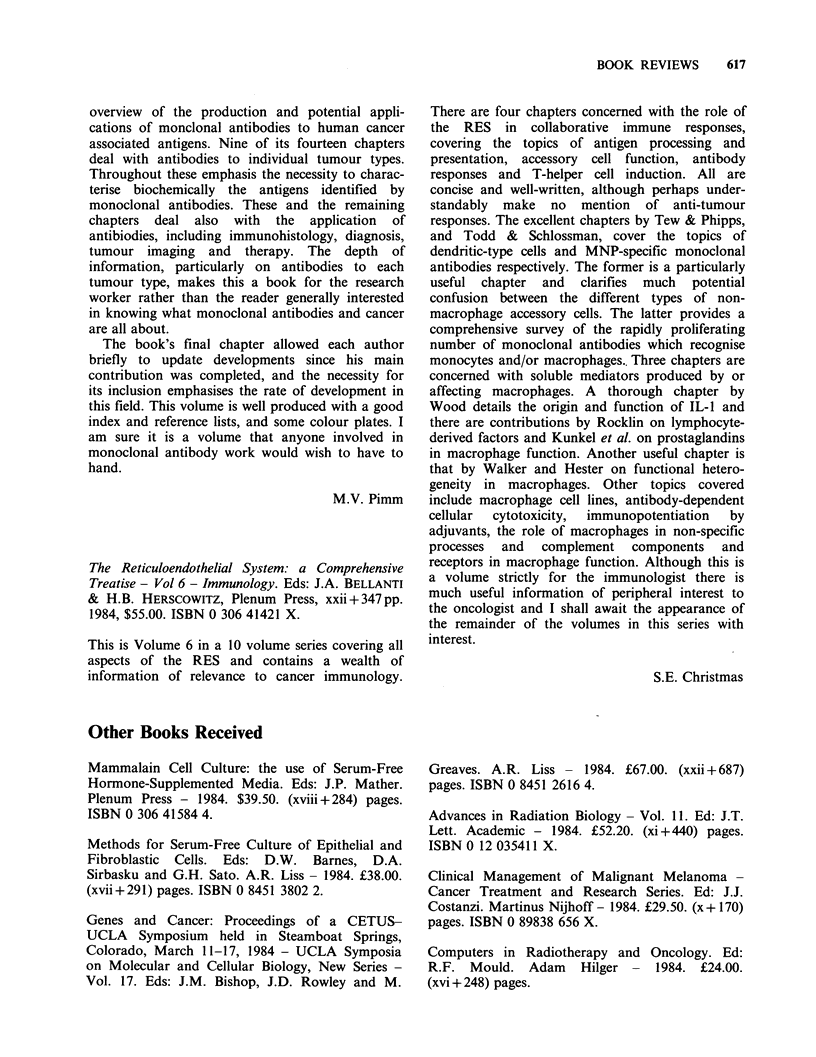# The Reticuloendothelial System: a Comprehensive Treatise

**Published:** 1985-04

**Authors:** S.E. Christmas


*The Reticuloendothelial System: a Comprehensive Treatise – Vol 6 – Immunology*. Eds: J.A. BELLANTI & H.B. HERSCOWITZ, Plenum Press, xxii + 347 pp. 1984, $55.00. ISBN 0 306 41421 X.

This is Volume 6 in a 10 volume series covering all aspects of the RES and contains a wealth of information of relevance to cancer immunology. There are four chapters concerned with the role of the RES in collaborative immune responses, covering the topics of antigen processing and presentation, accessory cell function, antibody responses and T-helper cell induction. All are concise and well-written, although perhaps understandably make no mention of anti-tumour responses. The excellent chapters by Tew & Phipps, and Todd & Schlossman, cover the topics of dendritic-type cells and MNP-specific monoclonal antibodies respectively. The former is a particularly useful chapter and clarifies much potential confusion between the different types of non-macrophage accessory cells. The latter provides a comprehensive survey of the rapidly proliferating number of monoclonal antibodies which recognise monocytes and/or macrophages.. Three chapters are concerned with soluble mediators produced by or affecting macrophages. A thorough chapter by Wood details the origin and function of IL-1 and there are contributions by Rocklin on lymphocyte-derived factors and Kunkel *et al.* on prostaglandins in macrophage function. Another useful chapter is that by Walker and Hester on functional heterogeneity in macrophages. Other topics covered include macrophage cell lines, antibody-dependent cellular cytotoxicity, immunopotentiation by adjuvants, the role of macrophages in non-specific processes and complement components and receptors in macrophage function. Although this is a volume strictly for the immunologist there is much useful information of peripheral interest to the oncologist and I shall await the appearance of the remainder of the volumes in this series with interest.

S.E. Christmas